# Four patients with a history of acute exacerbations of COPD: implementing the CHEST/Canadian Thoracic Society guidelines for preventing exacerbations

**DOI:** 10.1038/npjpcrm.2015.23

**Published:** 2015-05-07

**Authors:** Ioanna Tsiligianni, Donna Goodridge, Darcy Marciniuk, Sally Hull, Jean Bourbeau

**Affiliations:** 1 Agia Barbara Health Care Center, Heraklion, Crete, Greece; 2 Department of Thoracic Medicine, Clinic of Social and Family Medicine, University of Crete, Heraklion, Crete, Greece; 3 Department of Medicine, College of Medicine, University of Saskatchewan, Saskatoon, SK, Canada; 4 Division of Respirology, Critical Care and Sleep Medicine, University of Saskatchewan, Saskatoon, SK, Canada; 5 Centre for Primary Care and Public Health, Blizard Institute, Queen Mary University of London, London, UK; 6 Respiratory Epidemiology and Clinical Research Unit, Montreal Chest Institute, McGill University Health Centre, Montréal, QC, Canada

## Abstract

The American College of Chest Physicians and Canadian Thoracic Society have jointly produced evidence-based guidelines for the prevention of exacerbations in chronic obstructive pulmonary disease (COPD). This educational article gives four perspectives on how these guidelines apply to the practical management of people with COPD. A current smoker with frequent exacerbations will benefit from support to quit, and from optimisation of his inhaled treatment. For a man with very severe COPD and multiple co-morbidities living in a remote community, tele-health care may enable provision of multidisciplinary care. A woman who is admitted for the third time in a year needs a structured assessment of her care with a view to stepping up pharmacological and non-pharmacological treatment as required. The overlap between asthma and COPD challenges both diagnostic and management strategies for a lady smoker with a history of asthma since childhood. Common threads in all these cases are the importance of advising on smoking cessation, offering (and encouraging people to attend) pulmonary rehabilitation, and the importance of self-management, including an action plan supported by multidisciplinary teams.

## Case study 1: A 63-year-old man with moderate/severe COPD and a chest infection

A 63-year-old self-employed plumber makes a same-day appointment for another ‘chest infection’. He caught an upper respiratory tract infection from his grandchildren 10 days ago, and he now has a productive cough with green sputum, and his breathlessness and fatigue has forced him to take time off work.

He has visited his general practitioner with similar symptoms two or three times every year in the last decade. A diagnosis of COPD was confirmed 6 years ago, and he was started on a short-acting β_2_-agonist. This helped with his day-to-day symptoms, although recently the symptoms of breathlessness have been interfering with his work and he has to pace himself to get through the day. Recovering from exacerbations takes longer than it used to—it is often 2 weeks before he is able to get back to work—and he feels bad about letting down customers. He cannot afford to retire, but is thinking about reducing his workload.

He last attended a COPD review 6 months ago when his FEV_1_ was 52% predicted. He was advised to stop smoking and given a prescription for varenicline, but he relapsed after a few days and did not return for the follow-up appointment. He attends each year for his ‘flu vaccination’. His only other medication is an ACE inhibitor for hypertension.

### Managing the presenting problem. Is it a COPD exacerbation?

A COPD exacerbation is defined as ‘an acute event characterised by a worsening of the patient’s respiratory symptoms that is beyond normal day-to-day variation and leads to change in medications’.^1,2^ The worsening symptoms are usually increased dyspnoea, increased sputum volume and increased sputum purulence.^1,2^ All these symptoms are present in our patient who experiences an exacerbation triggered by a viral upper respiratory tract infection—the most common cause of COPD exacerbations. Apart from the management of the acute exacerbation that could include antibiotics, oral steroids and increased use of short-acting bronchodilators, special attention should be given to his on-going treatment to prevent future exacerbations.^2^ Short-term use of systemic corticosteroids and a course of antibiotics can shorten recovery time, improve lung function (forced expiratory volume in one second (FEV_1_)) and arterial hypoxaemia and reduce the risk of early relapse, treatment failure and length of hospital stay.^1,2^ Short-acting inhaled β_2_-agonists with or without short-acting anti-muscarinics are usually the preferred bronchodilators for the treatment of an acute exacerbation.^1^

### Reviewing his routine treatment

**Figure 1:**
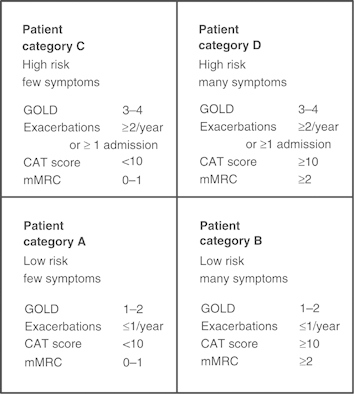
The four categories of COPD based on assessment of symptoms and future risk of exacerbations (adapted by Gruffydd-Jones,^5^ from the Global Strategy for Diagnosis, Management and Prevention of COPD).^1^ CAT, COPD Assessment Tool; COPD, chronic obstructive pulmonary disease; mMRC, modified Medical Research Council Dyspnoea Scale.

One of the concerns about this patient is that his COPD is inadequately treated. The Global Initiative for Chronic Obstructive Lung Disease (GOLD) suggests that COPD management be based on a combined assessment of symptoms, GOLD classification of airflow limitation, and exacerbation rate.^1^ The modified Medical Research Council (mMRC) dyspnoea score^3^ or the COPD Assessment Tool (CAT)^4^ could be used to evaluate the symptoms/health status. History suggests that his breathlessness has begun to interfere with his lifestyle, but this has not been formally asssessed since the diagnosis 6 years ago. Therefore, one would like to be certain that these elements are taken into consideration in future management by involving other members of the health care team. The fact that he had two to three exacerbations per year puts the patient into GOLD category C–D (see Figure 1) despite the moderate airflow limitation.^1,5^ Our patient is only being treated with short-acting bronchodilators; however, this is only appropriate for patients who belong to category A. Treatment options for patients in category C or D should include long-acting muscarinic antagonists (LAMAs) or long-acting β_2_-agonists (LABAs), which will not only improve his symptoms but also help prevent future exacerbations.^2^ Used in combination with LABA or LAMA, inhaled corticosteroids also contribute to preventing exacerbations.^2^

### Prevention of future exacerbations

**Figure 2:**
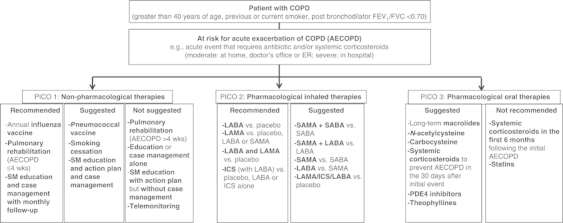
Decision tree for prevention of acute exacerbations of COPD (reproduced with permission from the CHEST/CTS Guidelines for the prevention of exacerbations in COPD).^2^ This decision tree for prevention of acute exacerbations of COPD is arranged according to three key clinical questions using the PICO format: non-pharmacologic therapies, inhaled therapies and oral therapies. The wording used is ‘Recommended or Not recommended’ when the evidence was strong (Level 1) or ‘Suggested or Not suggested’ when the evidence was weak (Level 2). CHEST/CTS, American College of Chest Physicians and Canadian Thoracic Society; COPD, chronic obstructive pulmonary disease; FEV_1_, forced expiratory volume in one second; FVC, forced vital capacity; LABA, long-acting β-agonist; LAMA, long-acting muscarinic antagonist; ICS, inhaled corticosteroids; SAMA, short-acting muscarinic antagonist; SABA, short-acting β-agonist; SM, self-management.

Exacerbations should be prevented as they have a negative impact on the quality of life; they adversely affect symptoms and lung function, increase economic cost, increase mortality and accelerate lung function decline.^1,2^ Figure 2 summarises the recommendations and suggestions of the joint American College of Chest Physicians and Canadian Thoracic Society (CHEST/CTS) Guidelines for the prevention of exacerbations in COPD.^2^ The grades of recommendation from the CHEST/CTS guidelines are explained in Table 1.

### Pharmacological approach

In patients with moderate-to-severe COPD, the use of LABA or LAMA compared with placebo or short-acting bronchodilators is recommended to prevent acute exacerbations (Grades 1B and 1A, respectively).^2,6,7^ LAMAs are associated with a lower rate of exacerbations compared with LABAs (Grade 1C).^2,6^ The inhaler technique needs to be checked and a suitable device selected. If our patient does not respond to optimizing inhaled medication and continues to have two to three exacerbations per year, there are additional options that offer pulmonary rehabilitation and other forms of pharmacological therapy, such as a macrolide, theophylline, phosphodieseterase (PDE4) inhibitor or *N*-acetylocysteine/carbocysteine,^2^ although there is no information about their relative effectiveness and the order in which they should be prescribed. The choice of prescription should be guided by the risk/benefit for a given individual, and drug availability and/or cost within the health care system.

### Non-pharmacological approach

A comprehensive patient-centred approach based on the chronic care model could be of great value.^2,8^

### This should include the following elements

- Vaccinations: the 23-valent pneumococcal vaccine and annual influenza vaccine are suggested as part of the overall medical management in patients with COPD.^2^ Although there is no clear COPD-specific evidence for the pneumococcal vaccine and the evidence is modest for influenza, the CHEST/CTS Guidelines concur with advice of the World Health Organization (WHO)^9^ and national advisory bodies,^10–12^ and supports their use in COPD patients who are at risk for serious infections.^2^
- Smoking cessation (including counselling and treatment) has low evidence for preventing exacerbations (Grade 2C).^2^ However, the benefits from smoking cessation are outstanding as it improves COPD prognosis, slows lung function decline and improves the quality of life and symptoms.^1,2,13,14^ Our patient has struggled to quit in the past; assessing current readiness to quit, and encouraging and supporting a future attempt is a priority in his care.
- Pulmonary rehabilitation (based on exercise training, education and behaviour change) in people with moderate-to-very-severe COPD, provided within 4 weeks of an exacerbation, can prevent acute exacerbations (Grade 1C).^2^ Pulmonary rehabilitation is also an effective strategy to improve symptoms, the quality of life and exercise tolerance,^15,16^ and our patient should be encouraged to attend a course.
- Self-management education with a written action plan and supported by case management providing regular direct access to a health care specialist reduces hospitalisations and prevents severe acute exacerbations (Grade 2C).^2^ Some patients with good professional support can have an emergency course of steroids and antibiotics to start at the onset of an exacerbation in accordance with their plan.

Finally, close follow-up is needed for our patient as he was inadequately treated, relapsed from smoking cessation after a few days despite varenicline, and missed his follow-up appointment. A more alert health care team may have been able to identify these issues, avoid his relapse and take a timely approach to introducing additional measures to prevent his recurrent acute exacerbations.

## Case study 2: A 74-year-old man with very severe COPD living alone in a remote community

A 74-year-old man has a routine telephone consultation with the respiratory team. He has very severe COPD (his FEV_1_ 2 years ago was 24% of predicted) and he copes with the help of his daughter who lives in the same remote community. He quit smoking the previous year after an admission to the hospital 50 miles away, which he found very stressful. He and his family managed another four exacerbations at home with courses of steroids and antibiotics, which he commenced in accordance with a self-management plan provided by the respiratory team.

His usual therapy consists of regular long-acting β_2_-agonist/inhaled steroid combination and a long-acting anti-muscarinic. He has a number of other health problems, including coronary heart disease and osteoarthritis and, in recent times, his daughter has become concerned that he is becoming forgetful. He manages at home by himself, steadfastly refusing social help and adamant that he does not want to move from the home he has lived in for 55 years.

This is a common clinical scenario, and a number of important issues require attention, with a view to optimising the management of this 74-year-old man suffering from COPD. He has very severe obstruction, is experiencing frequent acute flare-ups, is dependent and isolated and has a number of co-morbidities. To work towards preventing future exacerbations in this patient, a comprehensive plan addressing key medical and self-care issues needs to be developed that accounts for his particular context.

### Optimising medical management

According to the CHEST/CTS Guidelines for prevention of acute exacerbations of COPD,^2^ this patient should receive an annual influenza vaccination and may benefit from a 23-valent pneumococcal vaccine (Grades 1B and 2C, respectively). Influenza infection is associated with greater risk of mortality in COPD, as well as increased risk of hospitalisation and disease progression.^1^ A diagnosis of COPD also increases the risk for pneumococcal disease and related complications, with hospitalisation rates for patients with COPD being higher than that in the general population.^10,17^ Although existing evidence does not support the use of this vaccine specifically to prevent exacerbations of COPD,^1^ administration of the 23-valent pneumococcal vaccine is recommended as a component of overall medical management.^9–12^

Long-term oxygen therapy has been demonstrated to improve survival in people with chronic hypoxaemia;^18^ it would be helpful to obtain oxygen saturation levels and consider whether long-term oxygen therapy would be of benefit to this patient.

Even though this patient is on effective medications, further optimisation of pharmacologic therapy should be undertaken, including reviewing administration technique for the different inhaler devices.^19^ Maintenance PDE4 inhibitors, such as roflumilast or theophyllines, long-term macrolides (i.e., azithromycin) or oral *N*-acetylcysteine are potential considerations. Each of these therapeutic options has demonstrated efficacy in preventing future acute exacerbations, although they should be used with caution in this frail elderly man.^2^ This patient would benefit from a review of co-morbidities, including a chest X-ray, electrocardiogram, memory assessment and blood tests including haemoglobin, glucose, thyroid and renal function assessments.

### Pulmonary rehabilitation, supported self-management and tele-health care

Pulmonary rehabilitation for patients who have recently experienced an exacerbation of COPD (initiated <4 weeks following the exacerbation) has been demonstrated to prevent subsequent exacerbations (Grade 1C).^2^ Existing evidence suggests that pulmonary rehabilitation does not reduce future exacerbations when the index exacerbation has occurred more than 4 weeks earlier;^2^ however, its usefulness is evident in other important patient-centred outcomes such as improved activity, walking distance and quality of life, as well as by reduced shortness of breath. It would be appropriate to discuss this and enable our patient to enrol in pulmonary rehabilitation.

The patient’s access to pulmonary rehabilitation in his remote location, however, is likely to be limited. Several reports have noted that only one to two percent of people with COPD are able to access pulmonary rehabilitation programmes within Canada,^20^ the United States^21^ and the United Kingdom.^22^ Alternatives to hospital-based pulmonary rehabilitation programmes, such as home-based programmes or programmes offered via tele-health, may be options for this patient.^23^ Home-based pulmonary rehabilitation programmes have been found to improve exercise tolerance, symptom burden and quality of life.^24–27^ Outcomes of a pulmonary rehabilitation programme offered via tele-health have also been found to be comparable to those of a hospital-based programme,^28^ and may be worth exploring.

Written self-management (action) plans, together with education and case management, are suggested in the CHEST/CTS guidelines as a strategy to reduce hospitalisation and emergency department visits attributable to exacerbations of COPD (Grade 2B).^2^ Our patient has an existing action plan, which has enabled him and his family to manage some exacerbations at home. Although the patient has likely had some education on COPD and its management in the past, on-going reinforcement of key principles may be helpful in preventing future exacerbations.

The self-management plan should be reviewed regularly to ensure the advice remains current. The patient’s ability to use the self-management plan safely also needs to be assessed, given his daughter’s recent observation of forgetfulness and his living alone. Cognitive impairment is being increasingly recognised as a significant co-morbidity of COPD.^29,30^ Patients who were awaiting discharge from hospital following an exacerbation of COPD were found to perform significantly worse on a range of cognitive functional measures than a matched group with stable COPD, a finding that persisted 3 months later.^29^ Cognitive impairment may contribute independently to the risk for future exacerbations by increasing the likelihood of incorrect inhaler device use and failure to adhere to recommended treatments.^29^

Given that this patient resides in a remote location, access to case management services that assist in preventing future exacerbations may be difficult or impossible to arrange. Although there is currently insufficient evidence that in general the use of telemonitoring contributes to the prevention of exacerbations of COPD,^2^ tele-health care for this remotely located patient has potential to allow for case management at a distance, with minimal risk to the patient. Further study is needed to address this potential benefit.

### Assessing for and managing frailty

Recognising this patient’s co-morbid diagnoses of coronary heart disease and osteoarthritis, careful assessment of functional and self-care abilities would be appropriate. Almost 60% of older adults with COPD meet the criteria for frailty.^31^ Frailty is defined as a dynamic state associated with decline of physiologic reserves in multiple systems and inability to respond to stressful insults.^32^ Frailty is associated with an increased risk for institutionalisation and mortality.^33,34^ Given the complex needs of those who are frail, screening this patient for frailty would constitute patient-centred and cost-effective care. Frailty assessment tools, such as the seven-point Clinical Frailty Index,^35^ may provide structure to this assessment.

Admission to a hospital 50 miles away from our patient’s home last year for an exacerbation was stressful. Since his hospitalisation, this patient has experienced four additional exacerbations that have been managed at home in his remote community. It would be appropriate to explore the patient’s treatment wishes and determine whether the patient has chosen to refuse further hospitalisations. Our patient’s risk of dying is significant, with risk factors increasing the risk of short-term mortality following an exacerbation of COPD (GOLD Stage 4, age, male sex, confusion).^36^ Mortality rates between 22 and 36% have been documented in the first and second years, respectively, following an exacerbation,^37,38^ which also increase with the frequency and severity of hospitalisations.^39^

Our patient has refused social help and does not want to be relocated from his home. Ageing in their own home is a key goal of many older adults.^40,41^ Efforts to ensure that adequate resources to support the patient are available (and to support the daughter who is currently providing a lot of his care) will form an important part of the plan of care.

## Case study 3: A 62-year-old woman with severe COPD admitted with an exacerbation

A 62-year-old lady is admitted for the third time this year with an acute exacerbation of her severe COPD. Her FEV_1_ was 35% predicted at the recent outpatient visit. She retired from her job as a shop assistant 5 years ago because of her breathlessness and now devotes her time to her grandchildren who ‘exhaust her’ but give her a lot of pleasure.

She quit smoking 5 years ago. Over the years, her medication has increased, as nothing seemed to relieve her uncomfortable breathlessness, and, in addition to inhaled long-acting β_2_-agonist/ inhaled steroid combination and a long-acting anti-muscarinic, she is taking theophylline and carbocysteine, although she is not convinced of their beneficial effect. Oral steroid courses help her dyspnoea and she has taken at least six courses this year: she has an action plan and keeps an emergency supply of medication at home.

### A secondary care perspective on the management strategy for this woman

Acute exacerbations of COPD have serious negative consequences for health care systems and patients. The risk of future events and complications, such as hospital admission and poor patient outcomes (disability and reduced health status), can be improved through a combination of non-pharmacological and pharmacological therapies.^2^

### Evaluation of the patient, risk assessment and adherence to medication

The essential first step in the management of this lady (as for any patient) includes a detailed medical evaluation. Our patient has a well-established diagnosis of COPD with severe airflow obstruction (GOLD grade 3), significant breathlessness that resulted in her retiring from her job, and recurrent exacerbations. She does not have significant co-morbidity, although this requires to be confirmed. Further to the medical evaluation, it is important to assess her actual disease management (medication and proper use) as well as making sure she has adopted a healthy lifestyle (smoking cessation, physical activities and exercise). Does she live in a smoke-free environment? Effect of and evidence for smoking cessation in the prevention of acute exacerbations of COPD is low, but evidence exists for a reduction in cough and phlegm after smoking cessation and less lung function decline upon sustained cessation. With respect to the medication, never assume that it is taken as prescribed. When asking the patient, use open questions such as ‘I would like to hear how you take your medication on a typical day?’ instead of ‘Did you take the medication as prescribed’. Open questions tend to elicit more useful and pertinent information, and invite collaboration. Asking the patient to demonstrate her inhalation technique shows you the way she uses her different inhalation devices.

### Optimising the pharmacological therapy

The second step is to assess whether the patient is on optimal treatment to prevent exacerbations. In other words, can we do better helping the patient manage her disease and improving her well-being. As in the previous cases, vaccination, in particular, annual administration of the influenza vaccine, should be prescribed for this lady. We should evaluate other alternatives of pharmacological therapy that could improve symptoms, prevent exacerbations and reduce the use of repeated systemic corticosteroids with their important adverse effects (such as osteoporosis, cataracts, diabetes). Prescribing a PDE4 inhibitor (Grade 2A) or a long-term macrolide (Grade 2A) once a day would be a consideration for this lady.^2^ As there is no superiority trial comparing these two medications, our preference will be based on potential side effects, as well as cost and access to treatment. For PDE4 inhibitors, there are limited data on supplemental effectiveness in patients with COPD and chronic bronchitis concurrently using inhaled therapies, and they potentially have side effects such as diarrhoea, nausea, headache and weight loss. The side effects tend to diminish over time, but some patients may have to discontinue the therapy. Long-term macrolides have been studied in COPD patients already treated with inhaled therapies and shown to be effective, although clinicians need to consider in their individual patients the potential for harm, such as prolongation of the QT interval, hearing loss and bacterial resistance. Furthermore, the duration (beyond 1 year) and exact dosage of macrolide therapy (for example, once daily versus three times per week) are unknown.

### Making non-pharmacological therapy an essential part of the management

The third step, often neglected in the management of COPD patients, is non-pharmacological therapy. For this lady, we suggest self-management education with a written action plan and case management to improve how she deals with exacerbations (Grade 2B).^2^ The expectation will not be to reduce exacerbations but to prevent emergency department visits and hospital admissions. However, despite general evidence of efficacy,^42^ not all self-management interventions have been shown to be effective or to benefit all COPD patients^43,44^ (some have been shown to be potentially harmful^44^). The effectiveness of any complex intervention such as self-management in COPD critically depends on the health care professionals who deliver the intervention, as well on the patient and the health care system. The patient may not have the motivation or desire to change or to commit to an intensive programme. The individual patient’s needs, preferences and personal goals should inform the design of any intervention with a behavioural component. For this lady, it is essential to apply integrated disease management and to refer the patient to a pulmonary rehabilitation programme. Pulmonary rehabilitation has high value, including reducing the risk for hospitalisation in COPD patients with recent exacerbations (Grade 1C).^2^ The most important benefits our patient can expect from participating in structured supervised exercise within pulmonary rehabilitation are improved health status, exercise tolerance and a reduction in dyspnoea (Grade 1A).^2,15^ Pulmonary rehabilitation programmes provide clinicians with an opportunity to deliver education and self-management skills to patients with COPD, and are well established as a means of enhancing standard therapy to control and alleviate symptoms, optimise functional capacity and improve health-related quality of life.

## Case study 4: A 52-year-old lady with moderate COPD—and possibly asthma

A 52-year-old lady attends to discuss her COPD and specifically the problem she is having with exacerbations and time ‘off sick’. She is a heavy smoker, and her progressively deteriorating lung function suggests that she has moderate COPD, although she also has a history of childhood asthma, and had allergic rhinitis as a teenager. Recent spirometry showed a typical COPD flow-volume loop, although she had some reversibility (250 ml and 20%) with a post-bronchodilator FEV_1_ of 60% predicted.

She has a sedentary office job and, although she is breathless on exertion, this generally does not interfere with her lifestyle. The relatively frequent exacerbations are more troublesome. They are usually triggered by an upper respiratory infection and can take a couple of weeks to recover. She has had three exacerbations this winter, and as a result her employer is not happy with her sickness absence record and has asked her to seek advice from her general practitioner.

She has a short-acting β_2_-agonist, although she rarely uses it except during exacerbations. In the past, she has used an inhaled steroid, but stopped that some time ago as she was not convinced it was helping.

It is a welcome opportunity when a patient comes to discuss her COPD with a particular issue to address. With a history of childhood asthma, and serial COPD lung function tests, she has probably been offered many components of good primary care for COPD, but has not yet fully engaged with her management. We know that ~40% of people with COPD continue to smoke, and many are intermittent users of inhaled medications.^45^ It is easy to ignore breathlessness when both job and lifestyle are sedentary.

### Understanding her diagnosis and setting goals

Her readiness to engage can be supported by a move to structured collaborative care, enabling the patient to have the knowledge, resources and support to make the necessary changes. Much of this can be done by the primary care COPD team, including the pharmacist. Regular recall to maintain engagement is essential.

The combination of childhood asthma, rhinitis and a long history of smoking requires diagnostic review. This might include serial peak flows over 2 weeks to look for variability, and a chest X-ray, if not done recently, to rule out lung cancer as a reason for recent exacerbations. Her spirometry suggests moderate COPD,^1,46^ but she also has some reversibility, not enough to place her in the asthma camp but, combined with her past medical history, being enough to explore an asthma COPD overlap syndrome. This is important to consider as it may guide decisions on inhaled medication, and there is evidence that lung function deteriorates faster in this group.^47^ It is estimated that up to 20% of patients have overlap diagnoses, although the exact prevalence depends on the definition.^48^

### Reducing the frequency of exacerbations

Exacerbations in COPD are debilitating, often trigger hospital admission and hasten a progressive decline in pulmonary function.^2^ Written information on interventions that can slow down the course of COPD and reduce the frequency and impact of exacerbations will help to support progressive changes in management.

### Smoking cessation

Few people are unaware that cessation of smoking is the key intervention for COPD. Reducing further decline in lung function will slow down the severity of exacerbations. Finding a smoking cessation programme that suits her working life, exploring previous attempts at cessation, offering pharmacotherapy and a non-judgemental approach to further attempts at stopping are crucial.

### Immunisations

Many, but not all, exacerbations of COPD are triggered by viral upper respiratory tract infections. Annual flu immunisation is a part of regular COPD care and reduces exacerbations and hospitalisation when flu is circulating (Grade 1B). Pneumococcal immunisation should be offered, although evidence for reducing exacerbations is weak; those with COPD will be at greater risk for pneumococcal infection.^2^

### Pulmonary rehabilitation

**Figure 3:**
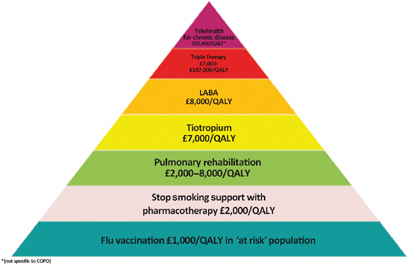
The COPD value pyramid (developed by the London Respiratory Network with The London School of Economics and reproduced with permission from the London Respiratory Team report 2013).^48^ This 'value' pyramid reflects what we currently know about the cost per QALY of some of the commonest interventions in COPD. It was devised as a tool for health care organisations to use to promote audit and to ensure adequate commissioning of non-pharmacological interventions. COPD, chronic obstructive pulmonary disease; LABA, long-acting β-agonist; QALY, quality-adjusted life-year.

Pulmonary rehabilitation improves symptoms, quality of life and reduces hospital admission.^49^ It is most efficacious in patients who are symptomatic (MRC dyspnoea scale 3 and above) and in terms of reducing exacerbations is most effective when delivered early after an exacerbation (Grade 1C).^2^ The major hurdle is encouraging patients to attend, with most programmes showing an attrition rate of 30% before the first appointment, and high rates of non-completion.^45,50^ Effective programmes that maintain the gains from aerobic exercise are more cost-effective than some of the inhaled medications in use (see Figure 3).^50^

### Medication

Inhaled medication is likely to improve our patient’s breathlessness and contribute to a reduction in exacerbation frequency. Currently, she uses only a short-acting β_2_-agonist. One wonders if she has a spacer? How much of the medicine is reaching her lungs? Repeated observation and training in inhaler use is essential if patients are to benefit from expensive medications.

With her history of asthma and evidence of some reversibility, the best choice of regular medication may be a combination of inhaled corticosteroid and a LABA. Guidelines suggest the asthma component in asthma COPD overlap syndrome should be the initial treatment target,^48^ and a LABA alone should be avoided. Warn about oral thrush, and the increased risk for pneumonia.^46^ If she chooses not to use an inhaled steroid, then a trial of a LAMA is indicated. Both drugs reduce exacerbation rates.^2,51^

Finally, ensuring early treatment of exacerbations speeds up recovery.^52^ Prescribe rescue medication (a 5–7-day course of oral steroids and antibiotic) to be started when symptomatic, and encourage attendance at a post-exacerbation review.

## Figures and Tables

**Table 1:** Summary of the grading system used in the CHEST/CTS guidelines for preventing exacerbations of COPD

*Strength of recommendation*	*Strength of evidence*	*Balance of benefits versus risk*	*Implication for clinicians*
1—Strong recommendation	A—High quality B—Moderate quality C—Low quality	Benefits clearly outweigh risks and burdens (or vice versa)	Strong recommendation, applies to most patients in most circumstances May change if higher quality evidence becomes available
2—Weak recommendation	A—High quality B—Moderate quality C—Low quality	Benefits closely balanced with risks and burden	Weak recommendation, best action may differ depending on circumstances Other alternatives may be equally reasonable

Abbreviations: CHEST/CTS, American College of Chest Physicians and Canadian Thoracic Society; COPD, chronic obstructive pulmonary disease.

Adapted from Guyatt *et al.* ^49^

## Notes

JB declares peer reviewed government grants (for conducting research in COPD self-management 'Living Well with COPD' and the longitudinal population-based Canadian Cohort Obstructive Lung Disease (CanCOLD) study) from Canadian Institute of Health Research Rx&D collaborative programme (Astra Zeneca, Boehringer- Ingelheim, GlaxoSmithKline, Merck, Nycomed, Novartis), Canadian Respiratory Research Network (CRRN), Respiratory Health Network of the FRQS and Research Institute of the MUHC. The remaining authors declare no conflict of interest.
